# Use of Modified Multiplier of the Simple Endoscopic Score for Crohn’s Disease Endoscopic Improvement Thresholds Enhances Effect Size Differentiation Between Adalimumab Versus Placebo: A Post Hoc Analysis of the EXTEND Trial

**DOI:** 10.1093/ecco-jcc/jjae171

**Published:** 2024-11-12

**Authors:** Emily C L Wong, Parambir S Dulai, John K Marshall, Stephen Laroux, Vipul Jairath, Walter Reinisch, Neeraj Narula

**Affiliations:** Department of Medicine (Division of Gastroenterology), Farncombe Family Digestive Health Research Institute, McMaster University, Hamilton, ON, Canada; Division of Gastroenterology, Northwestern University, Chicago, IL, USA; Department of Medicine (Division of Gastroenterology), Farncombe Family Digestive Health Research Institute, McMaster University, Hamilton, ON, Canada; AbbVie Bioresearch Center, Worcester, MA, USA; Department of Medicine, Division of Gastroenterology, Western University, London, ON, Canada; Department of Internal Medicine III, Division of Gastroenterology and Hepatology, Medical University of Vienna, Vienna, Austria; Department of Medicine (Division of Gastroenterology), Farncombe Family Digestive Health Research Institute, McMaster University, Hamilton, ON, Canada

**Keywords:** Crohn’s disease, SES-CD, MM-SES-CD, adalimumab

## Abstract

**Introduction:**

The Modified Multiplier of the Simple Endoscopic Score for Crohn’s Disease (MM-SES-CD) refines the assessment of endoscopic CD severity by differentially weighting parameters in the original SES-CD. A threshold of <22.5 for MM-SES-CD suggests endoscopic remission (ER) and correlates with a low risk of long-term disease progression. This study examines whether MM-SES-CD-defined ER and response criteria are more sensitive to treatment effects compared to conventional SES-CD definitions.

**Methods:**

This post hoc analysis of the EXTEND (extend the safety and efficacy of adalimumab through endoscopic healing) trial compared various SES-CD and MM-SES-CD definitions of ER and endoscopic response in CD patients treated with adalimumab or placebo. The study included participants with moderate–severe CD and a baseline MM-SES-CD score ≥ 22.5. The primary outcome of ER, defined as MM-SES-CD < 22.5, was evaluated at Weeks 12 and 52. Area under the curve (AUC) analyses compared thresholds for predicting Week 52 ER.

**Results:**

Of the 100 participants (77.5% of the EXTEND population), 51 received adalimumab and 49 received placebo. At Week 12, 62% achieved MM-SES-CD ≥ 20% reduction from baseline, compared to 39% with SES-CD ≥ 50% reduction. At Week 52, 56.9% of adalimumab-treated participants achieved MM-SES-CD < 22.5, compared to 10.2% in the placebo group. Modified Multiplier of the Simple Endoscopic Score for Crohn’s Disease ≥ 20% reduction at Week 12 better predicted Week 52 ER than SES-CD ≥ 50% reduction (AUC: 0.73 vs 0.62, *p* = 0.002).

**Conclusion:**

MM-SES-CD definitions improved discrimination between treatment and placebo and offered superior predictive accuracy for Week 52 ER. Its use may enhance trial efficiency and better predict long-term disease outcomes.

## Graphical Abstract

**Graphical Abstract:**
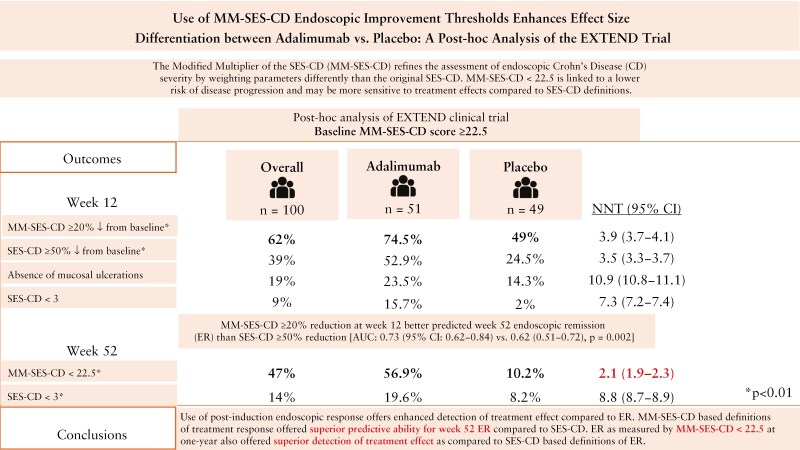

## 1. Introduction

Crohn’s disease (CD) is a chronic inflammatory condition of the gastrointestinal tract marked by mucosal ulcerations that may progress to irreversible intestinal damage.^1^ Accurate assessment of treatment efficacy is critical for optimizing patient outcomes and designing effective clinical trials. The Simple Endoscopic Score for Crohn’s Disease (SES-CD) is an endoscopic scoring tool that is commonly used in trials to evaluate endoscopic disease severity.^2^ However, the SES-CD has been criticized for its equal weighting of all parameters, which may limit its prognostic accuracy. Definitions of endoscopic remission (ER) with the SES-CD, which are used in trials, have also been criticized for their stringent criteria.

In recent years, the Modified Multiplier of the SES-CD (MM-SES-CD) has emerged as a more nuanced measure of quantifying endoscopic disease severity, offering potential advantages in assessing endoscopic disease severity and treatment response. The MM-SES-CD differentially weights individual SES-CD parameters based on its prognostic value for achieving ER.^3^ Compared to the SES-CD, the MM-SES-CD has demonstrated superior accuracy in predicting 1-year ER.

The EXTEND (extend the safety and efficacy of adalimumab through endoscopic healing) trial used the SES-CD to measure endoscopic severity in patients treated with adalimumab or placebo at Week 12 (post-induction) and Week 52, and defined ER as the absence of mucosal ulcerations.^4^ Using this definition, the trial missed its primary endpoint as no significant difference was shown between treatment groups at post-induction (27% for adalimumab vs 13% for placebo, *p* = 0.056). Previously, we demonstrated targets for MM-SES-CD reduction after-induction therapy (≥20% and ≥40% reduction from baseline) that were predictive of 1-year ER.^5^

This post hoc analysis of the EXTEND clinical trial aims to determine whether the endoscopic treatment effect measured using the MM-SES-CD is enhanced as compared to the SES-CD. We further explore the utility of the MM-SES-CD after-induction treatment in predicting post-maintenance ER compared to commonly used SES-CD measures.

## 2. Methods

### 2.1. Study design

This was a post hoc analysis of individual participant-level data from the EXTEND clinical trial (ClinicalTrials.gov identifier: NCT00348283). Data were used with permission from Abbvie Inc. and obtained through Vivli (Protocol #000010118). Details regarding the study design and eligibility criteria of EXTEND have been previously published.^6^ In short, adults with CD who had moderate to severely active CD based on a Crohn’s disease activity index (CDAI) score of 220-450 with evidence of mucosal ulcerations were eligible. All participants had prior TNF-alpha antagonist use or nonresponse to immunomodulators or glucocorticoids. At the beginning of the trial, all participants received adalimumab for 4 weeks and were subsequently randomized to continue receiving adalimumab 40 mg every other week or placebo. The study duration was 52 weeks with endoscopy performed at baseline, Weeks 12 and 52. All endoscopies were locally read using the SES-CD. Since it has previously been demonstrated that the target for ER using the MM-SES-CD is <22.5, this analysis was restricted to the 100 out of 129 participants (77.5%) from the EXTEND trial who had baseline MM-SES-CD scores ≥ 22.5.^7^ All SES-CD scores were converted into MM-SES-CD scores using the method outlined in previous publications.^8,9^

### 2.2. Outcomes

The primary outcome of this study was the proportion of participants who achieved ER, defined as MM-SES-CD < 22.5, at Week 12 among those with MM-SES-CD ≥ 22.5 at baseline. This cut-point was used based on previous analyses demonstrating the association of this threshold with lack of disease progression.^7^ Secondary outcomes included the proportion of participants who achieved ER based on other commonly used definitions (absence of mucosal ulcerations, SES-CD of 0, SES-CD < 3, SES-CD < 4) and endoscopic response (MM-SES-CD ≥ 20% reduction, MM-SES-CD ≥ 40% reduction, and SES-CD ≥ 50% reduction from baseline at Week 12). Outcomes of ER were also assessed at Week 52.

### 2.3. Statistical analysis

Demographic data of the study population were summarized using descriptive statistics. Categorical variables were summarized as proportions or percentages and compared using *t* tests. Means with standard deviations (SD) or medians with interquartile ranges (IQR) were used to describe continuous variables and compared using Chi-squared tests. All analyses were performed on an intention-to-treat basis. Sensitivity analyses were planned to evaluate outcomes among all 129 participants enrolled in EXTEND who received induction therapy and had endoscopic data available, regardless of MM-SES-CD score at baseline.

To assess the predictive performance of Week 12 MM-SES-CD and SES-CD on Week 52 outcomes, we conducted receiver operative characteristic (ROC) analyses and calculated the area under the curve (AUC). Area under the curve analyses were used to measure the accuracy of both scoring systems in predicting ER, providing a summary statistic that reflects the probability that a randomly chosen positive instance is ranked higher than a randomly chosen negative instance. In this study, AUC values were calculated for the prediction of achieving ER at Week 52, defined as an MM-SES-CD < 22.5. The AUC values were computed for both the overall population and stratified by treatment groups, adalimumab and placebo. These analyses were performed using ROC curves, which plot the true positive rate against the false positive rate at various threshold settings. Area under the curve values range from 0.5 (no discrimination) to 1.0 (perfect discrimination), with higher values indicating better predictive performance. DeLong’s test was used to compare AUCs and a *p* value < 0.05 was used to determine if statistically significant differences existed.

## 3. Results

Table 1 presents the baseline demographic characteristics of the study population, which included 100 participants with an MM-SES-CD score of 22.5 or higher at baseline. The mean age of participants was 37.9 years, with an SD of 12.2 years. The cohort was comprised of 62% females, with a similar distribution between the adalimumab (64.7%) and placebo (59.2%) groups. The mean duration of CD was 10.1 years. The median SES-CD and MM-SES-CD scores were 19 (IQR 13.5) and 37.3 (IQR 30.5-50), respectively, which were similar between treatment groups. Most participants had ileocolonic disease (65%) and the mean Crohn’s Disease Activity Index score at baseline was 324.8, indicating moderate to severe disease activity, with comparable scores between the adalimumab and placebo groups. Approximately half of the participants (52%) had prior exposure to anti-TNF therapy and concomitant corticosteroid use was reported among 31% of participants. Overall, baseline characteristics were similar between treatment groups.

**Table 1:** Baseline demographic characteristics of the study population (Modified Multiplier of the Simple Endoscopic Score for Crohn’s Disease score ≥ 22.5 at baseline).

	Overall (*n* = 100)	Adalimumab (*n* = 51)	Placebo (*n* = 49)
Age, mean (SD)	37.9 (12.2)	37.6 (11.9)	38.1 (12.7)
Female, *n* (%)	62 (62.0)	33 (64.7)	29 (59.2)
CD duration, mean (SD)	10.1 (8.2)	10.4 (8.1)	9.7 (8.2)
CD location, *n* (%)			
Colon	35 (35.0)	12 (23.5)	23 (46.9)
Ileocolonic	65 (65.0)	39 (76.5)	26 (53.1)
CDAI score, mean (SD)	324.8 (71.4)	328.7 (68.3)	320.7 (74.9)
SES-CD score, median (IQR)	19 (13.5-24)	17 (13-23)	20 (16-25)
MM-SES-CD score, median (IQR)	37.3 (30.5-50)	36.5 (30-50.5)	39.5 (30.5-49.5)
Prior anti-TNF use, *n* (%)	52 (52.0)	24 (47.1)	28 (57.1)
Concomitant corticosteroid use, *n* (%)	31 (31.0)	14 (27.5)	17 (34.7)

Table 2 demonstrates the proportion of participants with MM-SES-CD ≥ 22.5 at baseline achieving various definitions of ER at Week 12. More participants achieved MM-SES-CD ≥ 20% reduction from baseline than any other definition of ER or endoscopic response assessed (62/100, 62%). Compared to the placebo group, a significantly greater proportion of participants in the adalimumab-treated group achieved SES-CD < 3 [8/51 (15.7%) vs 1/49 (2%), *p* = 0.017)], SES-CD < 4 [15/51 (29.4%) vs 2/49 (4.1%), *p* = 0.001], SES-CD ≥ 20% reduction [32/51 (62.8%) vs 19/49 (38.8), *p* = 0.017], MM-SES-CD < 22.5 [33/51 (64.7%) vs 20/49 (40.8%), *p* = 0.017], MM-SES-CD ≥ 20% reduction [38/51 (74.5%) vs 24/49 (49%), *p* = 0.009], MM-SES-CD ≥ 40% reduction [33/51 (64.7%) vs 18/49 (36.7%), *p* = 0.005], and SES-CD ≥ 50% reduction from baseline [27/51 (52.9%) vs 12/49 (24.5%), *p* = 0.004]. No significant differences between groups were observed for ER defined as absence of mucosal ulcerations or SES-CD of 0 at Week 12. The highest number needed to treat (NNT) was observed for the outcome of SES-CD of 0 [NNT 12.8 (95% CI: 12.7-12.9)], whereas the lowest NNTs observed were for SES-CD ≥ 50% reduction from baseline [NNT 3.5 (95% CI: 3.3-3.7)], MM-SES-CD ≥ 40% reduction from baseline [NNT 3.6 (95% CI: 3.4-3.8)], and MM-SES-CD ≥ 20% reduction from baseline [NNT 3.9 (95% CI: 3.7-4.1)].

**Table 2:** Week 12 endoscopic remission among participants with Modified Multiplier of the Simple Endoscopic Score for Crohn’s Disease ≥ 22.5 at baseline (*n* = 100).

Endoscopic remission	Overall (*n* = 100)	Adalimumab (*n* = 51)	Placebo (*n* = 49)	*p* value (Adalimumab vs placebo)	Number needed to treat (95% CI)
Absence of mucosal ulcerations	19 (19.0)	12 (23.5)	7 (14.3)	0.239	10.9 (10.8-11.1)
SES-CD of 0	6 (6.0)	5 (9.8)	1 (2.0)	0.102	12.8 (12.7-12.9)
SES-CD < 3	9 (9.0)	8 (15.7)	1 (2.0)	0.017	7.3 (7.2-7.4)
SES-CD < 4	17 (17.0)	15 (29.4)	2 (4.1)	0.001	4 (3.9-4.1)
MM-SES-CD < 22.5	53 (53.0)	33 (64.7)	20 (40.8)	0.017	4.2 (4.0-4.4)
MM-SES-CD ≥ 20% reduction from baseline	62 (62.0)	38 (74.5)	24 (49.0)	0.009	3.9 (3.7-4.1)
MM-SES-CD ≥ 40% reduction from baseline	51 (51.0)	33 (64.7)	18 (36.7)	0.005	3.6 (3.4-3.8)
SES-CD ≥ 20% reduction from baseline	51 (51.0)	32 (62.8)	19 (38.8)	0.017	4.2 (4.1-4.3)
SES-CD ≥ 50% reduction from baseline	39 (39.0)	27 (52.9)	12 (24.5)	0.004	3.5 (3.3-3.7)

Sensitivity analyses were performed among 129 participants regardless of MM-SES-CD score at baseline (Supplementary Table S1). Similarly, MM-SES-CD ≥ 20% reduction from baseline had the highest proportion of participants achieving this threshold at Week 12 (77/129, 59.7%). Similar differences between treatment groups were observed for SES-CD < 3, SES-CD < 4, MM-SES-CD < 22.5, MM-SES-CD ≥ 20% reduction, MM-SES-CD ≥ 40% reduction, and SES-CD ≥ 50% reduction from baseline. A significantly greater proportion of participants treated with adalimumab achieved SES-CD of 0 [10/64 (15.6%) vs 2/65 (3.1%), *p* = 0.014]. Again, no significant difference was observed between groups for the outcome of absence of mucosal ulcerations.

Outcomes at Week 52 among participants with a baseline MM-SES-CD score of 22.5 or higher are presented in Table 3. A total of 10 participants achieved absence of mucosal ulcerations, all of whom were treated with adalimumab [10/51 (19.6%) vs 0/49, *p* < 0.001]. Simple Endoscopic Score for Crohn’s Disease of 0 was achieved among 8 participants (8.0%) overall, again all of whom were in the adalimumab group [8/51 (15.7%) vs 0/49, *p* < 0.001]. A total of 14 participants achieved SES-CD < 3, which was significantly greater among adalimumab-treated participants compared with placebo-treated participants [10/51 (19.6%) vs 4/49 (8.2%), *p* = 0.010]. Using the definition of SES-CD < 4, a total of 17 (17.0%) achieved this threshold, with a significantly higher proportion in the adalimumab group compared with the placebo group achieving this [13/51 (25.5%) vs 4/49 (8.2%), *p* = 0.021]. Modified Multiplier of the Simple Endoscopic Score for Crohn’s Disease < 22.5 was achieved by 47/100 (47.0%) overall, with a significant difference observed between the adalimumab group (29/51, 56.9%) and the placebo group (5/49, 10.2%) (*p* = 0.001). With this definition of ER, the lowest NNT was observed [NNT 2.1 (95% CI: 1.9-2.3)], which was statistically significantly lower than the NNTs for all other definitions of ER evaluated at Week 52. The highest NNT was observed for SES-CD < 3 with an NNT of 8.8 (95% CI: 8.7-8.9).

**Table 3:** Week 52 endoscopic remission among participants with Modified Multiplier of the Simple Endoscopic Score for Crohn’s Disease ≥ 22.5 at baseline (*n* = 100).

Endoscopic remission	Overall (*n* = 100)	Adalimumab (*n* = 51)	Placebo (*n* = 49)	*p* value (Adalimumab vs placebo)	Number needed to treat (95% CI)
Absence of mucosal ulcerations	10 (10.0)	10 (19.6)	0 (0)	<0.001	5.1 (5.0-5.2)
SES-CD of 0	8 (8.0)	8 (15.7)	0 (0)	<0.001	6.4 (6.3-6.5)
SES-CD < 3	14 (14.0)	10 (19.6)	4 (8.2)	0.010	8.8 (8.7-8.9)
SES-CD < 4	17 (17.0)	13 (25.5)	4 (8.2)	0.021	5.8 (5.7-5.9)
MM-SES-CD < 22.5	47 (47.0)	29 (56.9)	5 (10.2)	0.001	2.1 (1.9-2.3)

To better understand the potential impact of disease location on differences observed between ER defined using the SES-CD versus MM-SES-CD, stratified analyses were performed based on disease location. By Week 52, a total of 33 participants achieved MM-SES-CD < 22.5 but did not achieve SES-CD < 3 (19 in the adalimumab-treated group and 14 in the placebo-treated patients). In the adalimumab-treated group, 5/19 (26.3%) had disease in the colon only at baseline, of which 1/5 (25%) had mild endoscopic disease (SES-CD < 6). In addition, 14/19 (73.7%) had disease in the ileum and colon at baseline, of which 4/14 (28.6%) participants had mild endoscopic disease activity (SES-CD < 6). Similar differences were also observed with the placebo group, where a total of 6/14 (42.9%) had disease in the colon, of which 2/6 (33.3%) had mild endoscopic disease activity in contrast to 8/14 (57.1%), who had disease in the ileum and colon, of which 2/8 (25%) had mild endoscopic disease activity.

The predictive ability of Week 12 MM-SES-CD versus SES-CD for achieving Week 52 ER (MM-SES-CD < 22.5) was evaluated among participants with an MM-SES-CD score ≥ 22.5 at baseline (Table 4). Modified Multiplier of the Simple Endoscopic Score for Crohn’s Disease < 22.5 was selected as the definition for Week 52 ER as it demonstrated the lowest NNT and due to its association with lack of longer-term disease progression. Compared to other definitions of ER at Week 12, MM-SES-CD ≥ 20% reduction from baseline had the strongest ability to predict Week 52 ER [AUC: 0.73 (95% CI: 0.62-0.84)]. Other endoscopic improvement definitions using the MM-SES-CD had similar predictive ability at week 12 for week 52 ER, including MM-SES-CD < 22.5 [AUC: 0.71 (95% CI: 0.50-0.81)] and MM-SES-CD ≥ 40% reduction from baseline [AUC: 0.69 (95% CI: 0.59-0.80)]. By contrast, SES-CD thresholds performed comparatively worse. Lower AUCs were seen with all SES-CD thresholds, including SES-CD ≥ 50% reduction from baseline [AUC: 0.62 (95% CI: 0.51-0.72)], SES-CD of 0 [AUC: 0.54 (95% CI: 0.51-0.57)], SES-CD < 3 [AUC: 0.56 (95% CI: 0.52-0.59)], and SES-CD < 4 [AUC: 0.61 (0.56-0.65)]. Absence of mucosal ulcerations at Week 12 also did not perform as well as MM-SES-CD thresholds [AUC: 0.62 (95% CI: 0.57-0.67)]. Overall, findings were similar between adalimumab and placebo-treated patients.

**Table 4:** Predictive ability of Week 12 Modified Multiplier of the Simple Endoscopic Score for Crohn’s Disease (MM-SES-CD) versus Simple Endoscopic Score for Crohn’s Disease (SES-CD) on Week 52 endoscopic remission (MM-SES-CD < 22.5) among participants with MM-SES-CD ≥ 22.5 at baseline (*n* = 100).

Endoscopic remission at Week 12	AUC (95% CI)		
	Overall (*n* = 100)	Adalimumab (*n* = 51)	Placebo (*n* = 49)
Absence of mucosal ulcerations	0.62 (0.57-0.67)	0.63 (0.56-0.69)	0.61 (0.54-0.68)
SES-CD of 0	0.54 (0.51-0.57)	0.55 (0.51-0.60)	0.52 (0.49-0.55)
SES-CD < 3	0.56 (0.52-0.59)	0.58 (0.53-0.64)	0.52 (0.49-0.55)
SES-CD < 4	0.61 (0.56-0.65)	0.66 (0.59-0.72)	0.53 (0.49-0.57)
MM-SES-CD < 22.5	0.71 (0.60-0.81)	0.67 (0.33-1.00)	0.68 (0.55-0.81)
MM-SES-CD ≥ 20% reduction from baseline	0.73 (0.62-0.84)	0.72 (0.39-1.00)	0.69 (0.56-0.83)
MM-SES-CD ≥ 40% reduction from baseline	0.69 (0.59-0.80)	0.67 (0.33-1.00)	0.65 (0.52-0.77)
SES-CD ≥ 50% reduction from baseline	0.62 (0.51-0.72)	0.60 (0.27-0.94)	0.55 (0.43-0.67)

Table 5 compares the AUCs for Week 12 ER definitions of MM-SES-CD versus SES-CD. Compared to SES-CD ≥ 50% reduction from baseline, MM-SES-CD ≥ 20% reduction and MM-SES-CD ≥ 40% reduction predicted Week 52 ER with significantly greater accuracy (*p* = 0.002 and *p* < 0.001, respectively). MM-SES-CD < 22.5 at Week 12 also had significantly greater accuracy for predicting Week 52 ER compared to all definitions of SES-CD assessed, including SES-CD of 0, SES-CD < 4, and SES-CD < 3 (*p* < 0.001 for all comparisons). Findings were consistent among adalimumab and placebo groups.

**Table 5:** Comparison of area under the curves for Week 12 endoscopic remission of Modified Multiplier of the Simple Endoscopic Score for Crohn’s Disease versus Simple Endoscopic Score for Crohn’s Disease.

Endoscopic remission at Week 12	Overall (*n* = 100)	*p* value	Adalimumab (*n* = 51)	*p* value	Placebo (*n* = 49)	*p* value
MM-SES-CD ≥ 20% reduction from baseline	0.73 (0.62-0.84)	0.002	0.72 (0.39-1.00)	<0.001	0.69 (0.56-0.83)	0.006
SES-CD ≥ 50% reduction from baseline	0.62 (0.51-0.72)		0.60 (0.27-0.94)		0.55 (0.43-0.67)	
MM-SES-CD ≥ 40% reduction from baseline	0.69 (0.59-0.80)	<0.001	0.67 (0.33-1.00)	0.010	0.65 (0.52-0.77)	0.008
SES-CD ≥ 50% reduction from baseline	0.62 (0.51-0.72)		0.60 (0.27-0.94)		0.55 (0.43-0.67)	
MM-SES-CD < 22.5	0.71 (0.60-0.81)	<0.001	0.67 (0.33-1.00)	<0.001	0.68 (0.55-0.81)	<0.001
SES-CD of 0	0.54 (0.51-0.57)		0.55 (0.51-0.60)		0.52 (0.49-0.55)	
MM-SES-CD < 22.5	0.71 (0.60-0.81)	<0.001	0.67 (0.33-1.00)	0.648	0.68 (0.55-0.81)	<0.001
SES-CD < 4	0.61 (0.56-0.65)		0.66 (0.59-0.72)		0.53 (0.49-0.57)	
MM-SES-CD < 22.5	0.71 (0.60-0.81)	<0.001	0.67 (0.33-1.00)	<0.001	0.68 (0.55-0.81)	<0.001
SES-CD < 3	0.56 (0.52-0.59)		0.58 (0.53-0.64)		0.52 (0.49-0.55)	

## 4. Discussion

In this post-hoc analysis, we demonstrated the clinical utility of the MM-SES-CD for the assessment of endoscopic endpoints in clinical trials for CD. Specifically, we found that Week 12 ER defined as MM-SES-CD < 22.5 would have identified a significant difference between adalimumab- and placebo-treated groups in the EXTEND study among those with an MM-SES-CD ≥ 22.5 at baseline. Instead, the EXTEND study defined ER using the more stringent criterion of absence of mucosal ulcerations, which failed to identify a significant difference between groups at Week 12. We also identified that Week 12 ER defined as SES-CD < 4 would have also demonstrated a difference between groups, which we previously suggested could be a target SES-CD score for ER within clinical trials of CD.^7^ Finally, we demonstrated that MM-SES-CD ≥ 20% reduction from baseline at Week 12 had the strongest predictive ability for subsequent ER at Week 52.

The original EXTEND study showed a significant difference between adalimumab and placebo-treated participants at Week 52 when ER was defined as the absence of mucosal ulcerations. However, this definition has been commonly criticized for its stringency (ie, a binary measure anchored on a single disease feature), as evidenced by the low number of participants who achieved this outcome. Recent findings from the REACT-2 trial demonstrated that one-third of participants with CD could only achieve this outcome.^10^ Previous studies have also assessed alternate SES-CD cut-points using EXTEND data, which have demonstrated that greater stringency of endoscopic endpoint definitions reduces the ability to detect changes in endoscopic disease severity.^11^ By contrast, 47% of total participants achieved MM-SES-CD < 22.5 at Week 52, with a 46% difference observed in achievement of this outcome between adalimumab- and placebo-treated participants (56.9% vs 10.2%).

At Week 52, we demonstrated that MM-SES-CD < 22.5 was best able to discriminate treatment effect, as indicated by the low NNT of 2.1 that was observed with this definition. The NNTs observed with the Week 52 outcome of MM-SES-CD < 22.5 were dramatically lower than what was observed with conventional SES-CD-based definitions for ER (eg, compared to SES-CD < 3, NNT 2.1 vs 8.8, and compared to SES-CD of 0, NNT 2.1 vs 6.4). Similarly, SES-CD < 4 was achieved among 17% of participants, with a 17% delta between groups (25.5% vs 8.2%, NNT 5.8 (5.7-5.9)). Therefore, MM-SES-CD < 22.5 may be a more pragmatic target for ER at Week 52 based on the increased number of participants who can achieve this outcome, and the larger effect size that was observed in drug-treated compared with placebo-treated participants.

It is worth noting that in our study, MM-SES-CD improvements by Week 12 generally outperformed the SES-CD cutoffs we evaluated for predicting MM-SES-CD < 22.5 at Week 52. This is likely due to the inclusion of patients with minimal endoscopic inflammation who still achieved MM-SES-CD < 22.5. Simple Endoscopic Score for Crohn’s Disease definitions such as SES-CD < 3, SES-CD of 0, or the absence of mucosal ulcerations exclude patients with erosions or ulcers throughout the ileum and colon. As a result, some patients who experience meaningful reductions in their endoscopic burden at Week 12 and meet the MM-SES-CD < 22.5 threshold at Week 52 are not captured by these stricter SES-CD criteria. We have previously demonstrated that attaining MM-SES-CD < 22.5 at 1-year after initiation of advanced therapy was associated with a lack of disease progression so early assessments that predict Week 52 MM-SES-CD < 22.5 are clinically relevant.^3^

The insights from this analysis are crucial for future trial design. Understanding the expected changes in MM-SES-CD scores post-induction and their predictive value for long-term outcomes can inform sample size calculations and outcome expectations. By using MM-SES-CD < 22.5 as a target endpoint, trialists can expect a larger number of drug-treated patients to achieve this outcome than what is observed when using various SES-CD targets. Furthermore, the large deltas observed in drug- compared to placebo-treated patients when using the MM-SES-CD also may help clinical trialists demonstrate significant differences in endoscopic efficacy with smaller sample sizes than needed when using more stringent definitions with the SES-CD. This may prove particularly meaningful in phase 2 trials where smaller sample sizes are often used, and signals of treatment efficacy are needed to pursue large expensive phase 3 trials. Our findings reinforce the importance of reassessing endoscopic outcomes by regulatory agencies, such as the Federal Drug Administration (FDA), to better reflect disease activity.^12^

Strengths of this study include use of individual participant-level data from the EXTEND clinical trial with locally read endoscopic evaluations. The comparative analysis between MM-SES-CD and traditional SES-CD measures adds value by demonstrating that MM-SES-CD performs at least comparable to well-established SES-CD thresholds at Week 12, and perhaps superior at Week 52. This supports its clinical utility as an endoscopic endpoint in clinical trials. In addition, the large deltas observed in drug- compared to placebo-treated patients using the MM-SES-CD offers insights for trial design, which can help set realistic expectations and improve sample size calculations based on anticipated treatment effect.

However, there are limitations of our study that are worth noting. The generalizability of our results is limited by the use of data from a single trial with a small sample size. Further studies with larger sample sizes are necessary to confirm these results and ensure they apply across diverse patient populations. In addition, EXTEND assessed the efficacy of adalimumab. Additional studies are needed among participants treated with other therapies for CD to ensure broader applicability of our findings. While the study highlights the feasibility of using MM-SES-CD < 22.5 and SES-CD < 4 as ER targets at post-induction and through 52 weeks, it is unclear how well these definitions perform beyond Week 52. Finally, most of our study population had ileocolonic disease (65%), and it is plausible that observed differences between the SES-CD and MM-SES-CD could be attributed to disease location, particularly in the ileum where healing poses a greater challenge. However, we observed that participants with ileocolonic disease had similar rates of persistent mild endoscopic disease activity as those with disease limited to the colon only. Further research is needed to validate these thresholds across larger populations and assess their implications for longer-term patient outcomes and clinical practice.

In conclusion, this post hoc analysis underscores the clinical utility of the MM-SES-CD in assessing post-induction endpoints and predicting long-term ER in CD. This study highlights that the MM-SES-CD can be used as an endoscopic scoring tool within clinical trials of CD. Use of the endpoint MM-SES-CD < 22.5 is feasible within clinical trials, and large treatment differences between drug- and placebo-treated patients were observed in this trial when using the MM-SES-CD targets and thresholds. Clinical trials are very expensive to run, and performing several endoscopies on patients within the setting of a clinical trial is one of the main cost drivers. Ultimately, use of the MM-SES-CD may permit the detection of treatment effect with smaller sample sizes, which should reduce the possibility of type II error and could lead to cost savings as well. Future research should aim to validate these results in diverse settings and explore the clinical significance of different ER thresholds to further optimize the assessment of therapies in CD.

## Supplementary Data

Supplementary data are available online at *ECCO-JCC* online.

jjae171_suppl_Supplementary_Table_S1

## Data Availability

This publication (Vivli protocol #000010118) is based on research using data from Abbvie Inc. that has been made available through Vivli, Inc. Vivli has not contributed to or approved, and is not in any way responsible for, the contents of this publication.

## Notes

Guarantor of the article: Neeraj Narula
