# Supplementary material for: Use of Modified Multiplier of the Simple Endoscopic Score for Crohn’s Disease Endoscopic Improvement Thresholds Enhances Effect Size Differentiation Between Adalimumab Versus Placebo: A Post Hoc Analysis of the EXTEND Trial
Source: J Crohns Colitis. 2024 Nov 12;19(4):jjae171. doi: 10.1093/ecco-jcc/jjae171 (PMC12001330; doi:10.1093/ecco-jcc/jjae171)
Supplement: jjae171_suppl_Supplementary_Table_S1 [file jjae171_suppl_supplementary_table_s1.docx]

Supplementary Table 1 – Week 12 endoscopic remission among all participants (n=129)

| **Endoscopic Remission** | **Overall (n=129)** | **Adalimumab (n=64)** | **Placebo (n=65)** | **p-value (Adalimumab vs. placebo)** |
| --- | --- | --- | --- | --- |
| Absence of mucosal ulcerations | 28 (21.7) | 18 (28.1) | 10 (15.4) | 0.079 |
| SES-CD of 0 | 12 (9.3) | 10 (15.6) | 2 (3.1) | 0.014 |
| SES-CD < 3 | 17 (13.2) | 14 (21.9) | 3 (4.6) | 0.004 |
| SES-CD < 4 | 28 (21.7) | 24 (37.5) | 4 (6.2) | <0.001 |
| MM-SES-CD < 22.5 | 71 (55.0) | 44 (68.8) | 27 (41.5) | 0.002 |
| MM-SES-CD ≥20% reduction from baseline | 77 (59.7) | 48 (75.0) | 29 (44.6) | <0.001 |
| MM-SES-CD ≥40% reduction from baseline | 64 (49.6) | 42 (65.6) | 22 (33.9) | <0.001 |
| SES-CD ≥ 50% reduction from baseline | 51 (39.5) | 35 (54.7) | 16 (24.6) | <0.001 |
